# miRNA-146 negatively regulates the production of pro-inflammatory cytokines via NF-κB signalling in human gingival fibroblasts

**DOI:** 10.1186/s12950-014-0038-z

**Published:** 2014-12-06

**Authors:** Yu-feng Xie, Rong Shu, Shao-yun Jiang, Zhong-chen Song, Qiu-man Guo, Jia-chen Dong, Zhi-kai Lin

**Affiliations:** Department of Periodontology, College of Stomatology, Ninth People’s Hospital, School of Medicine, Shanghai Jiao Tong University, Shanghai Key Laboratory of Stomatology, 639 Zhi Zao Ju Road, Shanghai, 200011 China; Shanghai Key Laboratory of Stomatology and Shanghai Research Institute of Stomatology, Shanghai, China

**Keywords:** miRNA-146, Human gingival fibroblasts, Pro-inflammatory cytokines, NF-κB

## Abstract

**Objective:**

In human gingival fibroblasts (HGFs), TLR4 recognises Pathogen-associated molecular patterns (PAMPs), such as LPS, and subsequently activates downstream signals that lead to the production of pro-inflammatory cytokines. The aim of this study was to explore the mechanisms of LPS-induced miRNA-146 regulation of TLR4 signals in HGFs.

**Materials and methods:**

HGFs were treated with *Porphyromonas gingivalis* (*P.g*) LPS, the cells were harvested, and kinase phosphorylation levels were detected by western blot. Selective pharmacological inhibitors and agonists were used to block or activate the relevant kinases, miRNA-146a/b expression levels were detected by real-time PCR, and IL-1, IL-6, and TNF-α production were measured by enzyme-linked immunosorbent assays (ELISA). A luciferase reporter plasmid containing miRNA-146a/b promoter was tested in terms of p50/p65 regulation.

**Results:**

After *P.g* LPS treatment, NF-κB and Erk1/2 were strongly activated in HGFs. miRNA-146a/b, IL-1, IL-6 and TNF-α levels were down-regulated when NF-κB inhibitor was used. p50/p65 strongly activated miRNA-146a/b promoter as measured with the luciferase assay.

**Conclusion:**

In TLR4 signalling in HGFs, both miRNA-146a and miRNA-146b are downstream targets of NF-κB, but not of AP-1 signalling. miRNA-146a/b expression was specifically dependent on NF-κB but not Erk1/2 or JNK signalling.

## Introduction

Periodontal disease is one of the most common oral diseases. The third China National Oral Health Survey found that the overall prevalence of periodontal disease in the 35-44-year-old and 65-74-year-old Chinese populations exceeded 85% [1]. Toll-like receptor 4 (TLR4) is responsible for the recognition of distinct bacterial cell-wall components, such as LPS, and signal transduction [2]. LPS first binds to the cluster of differentiation-14 (CD14) receptor via the LPS-binding protein (LBP) and is then transferred to TLR4. Thereafter, the myeloid differentiation factor 88 (MyD88) adaptor protein links TLR4 to the interleukin-1 receptor-associated kinase-4 (IRAK4), which induces the phosphorylation of IRAK1. Tumour necrosis factor receptor-associated factor-6 (TRAF6) is also recruited to the receptor complex via association with phosphorylated IRAK1. TRAF6 transduces the signal through the TGF-β-activated kinase-1 (TAK1), TAK1-binding protein-1 (TAB1) and TAK1-binding protein-2 (TAB2) complexes, phosphorylates IκB kinase 1 (IKK1) and IκB kinase 2 (IKK2), and finally ubiquitinates inhibitor of NF-κB (IκB) and drives p65/p50 to translocate into the nucleus [3]. Simultaneously, TLR4 activates c-Jun N-terminal kinase (JNK) and extracellular signal-regulated kinase (Erk), which leads to the activation of activating protein-1 (AP-1), which ultimately results in the production of inflammatory cytokines such as IL-1β, IL-6 and TNF-α. It has been reported that LPS stimulation can also induce the phosphorylation of p44 and p42 (Erk1 and Erk2, respectively) [4] and the expression of c-jun and c-fos [5] in human gingival fibroblasts (HGFs). Understanding the molecular mechanisms by which LPS-TLR4 signalling is regulated in periodontal cells will aid the design of effective strategies for the diagnosis and treatment of human periodontal diseases.

miRNAs are short (18–25 nucleotides long) non-coding RNAs that regulate gene expression by binding to the 3’-untranslated region (UTR) of the mRNAs of target genes [6]. miRNAs were first discovered in 1993 in *Caenorhabditis elegans* [7]. miRNAs are important post-transcriptional regulators of diverse biological processes, such as development, tumourigenesis, inflammation, and infection [8]. Earlier research found that miR-146a is strongly elevated in LPS-stimulated human monocytic THP-1 cells via an NF-κB-dependent pathway, and thus miR-146 is considered as an important repressor of LPS-induced signalling via its targeting of IRAK1 and TRAF6 [9]. miR-146 also plays an important role in regulating IL-1β-induced cytokine production in human alveolar epithelial cell [10]. This function has also been reported in VSV (Vesicular Stomatitis Virus) -infected macrophages, and IRAK2 has been found to be a new target of miR-146a [11]. Together, these findings suggest that miR-146 has an important role in negative regulatory loop of LPS-TLR4 signalling in diverse cell types.

Given that different cell types have different cellular environments, the behaviours of miRNAs are widely diverse across distinct cellular environments. Although the LPS-TLR4 signal is important in HGFs, whether miRNAs, and if so which miRNAs, play key regulating roles remains obscure. In our previous studies [12], we found that miR-146a and miR-146b are highly expressed in inflammatory gingival tissues compared to healthy tissues. We also confirmed that miR-146 plays a critical role in down-regulating inflammatory cytokines in HGFs by targeting IRAK1 but not TRAF6, which implies that the behaviour of miR-146 in HGFs is unique. Based on these findings, we further identified a precise method for controlling miR-146 expression in HGFs. This approach employs pharmacological methods to block the activities of up- (IRAK1/4) and down-stream (IκB, JNK, and Erk) regulators of miR-146 with the aim of completely mapping the molecular regulation miR-146 to provide a drug design strategy based on miR-146 as a microRNA therapeutics for clinical trials.

## Materials and methods

### HGF cell culture

HGFs were prepared from explants of the gingiva of 10 periodontitis patients who were acquired during periodontal flap surgery after receiving the informed consent of the patients. The epithelial tissues were torn from the gingiva after 24 h of soaking in 2 U/ml dispase II (Takara, Japan). Gingival connective tissues were cut into pieces and cultured in Dulbecco’s modified Eagle’s medium (DMEM) (Gibco, USA) with 20% foetal bovine serum (FBS) (Hyclone, USA). The medium was changed every 3 days for total 10–20 days until confluent cell monolayers were formed [13]. After four or five subcultures, homogeneous, slim, spindle-shaped cells were obtained and cultured in DMEM with 10% FBS, penicillin (100 U/ml) and streptomycin sulphate (50 μg/ml). TPCA-1 (an IKK-2 inhibitor), PD98059 (a MEK-1/2 inhibitor), SP600125 (a JNK-1/2 inhibitor) or an IRAK1/4 inhibitor was added to the culture at the concentrations and times indicated below, with or without simulation of 1 μg/ml of *P.g* LPS (Ultrapure, activates TLR4 only, InvivoGen, USA). All inhibitors were purchased from Sigma (USA). Betulinic acid was purchased from R&D (USA).

### miRNA analysis

Total RNA was extracted from cultured cells with TRIzol (Invitrogen, USA) according to the manufacturer’s instructions. miRNA was polyadenylated and reverse transcribed using poly(A) polymerase and MMLV reverse transcriptase (Clontech, USA). miR-146a and miR-146b expressions in the total RNA extracts were measured with SYBR Advantage qPCR Premix (Clontech, USA), and the reactions were run on an ABI PRISM 7900HT Sequence Detection System (Applied Biosystems, USA). The U6 small nuclear RNA (NR_003027) was used as an internal control. Each sample was amplified in triplicate. Data were analysed with SDS software (ABI, USA). The sequences of the target mature miRNAs and specific forward PCR primers were follows: hsa-miR-146a, GGGTGAGAACTGAATTCCA; hsa-miR-146b-5p, GGGTGAGAACTGAATTCCA; universal reverse PCR primer, CAGTGCGTGTCGTGGAGT; and U6 small nuclear RNA primers, GCTTCGGCAGCACATATACTAAAAT (U6-forward) and CGCTTCACGAATTTGCGTGTCAT (U6-reverse).

### Luciferase assay

Two kilobases promoter sequence of miR-146a/b primary transcripts were cloned into the 5’ site of the luc2 reporter gene in pGL4.10 plasmid (Promega, USA). We co-transfected 200 ng of reporter plasmid, 20 ng of pRL-TK-Renillaluciferase and 100 ng of p50/p65 pcDNA3.0 plasmid into the HGFs using Lipofectamine 2000 (Invitrogen, USA). After 24 h of transfection, luciferase activity was measured using the Dual-Luciferase Reporter Assay System (Promega, USA) according to the manufacturer’s instructions. The luciferase data were normalised to transfection efficiency by dividing the firefly luciferase activity by the activity of Renilla luciferase.

### Western blot assay

A total of 10^5^ HGFs per sample were harvested and lysed with 100 μl lysis buffer (50 mM HEPES, pH 7.0), 1% Nonidet P-40, 5 mM EDTA, 450 mM NaCl, 10 mM Na pyrophosphate, and 50 mM NaF and freshly supplemented with inhibitors (1 mM Na orthovanadate, 1 mM PMSF, 10 μg/ml aprotinin, leupeptin, pepstatin) at room temperature for 20 min. Aliquots of the whole-cell extracts were prepared and subjected to 10% SDS-PAGE and then electroblotted onto nitrocellulose membranes. The membranes were then probed with Abs as indicated. Antibodies against p-JNK (Thr183/Tyr185), total JNK (56G8), p-Erk1/2 (Thr202/Tyr204, D13.14.4E), total Erk1/2 (137 F5), p-IRAK4 (Thr345/Ser346), total IRAK4, total IκB-α and HRP-labelled secondary antibody were purchased from Cell Signaling. Membranes were visualised with Super Signal West Pico Chemiluminescent Substrate (Pierce, USA)

### ELISA assay

For analysis of cytokine production in the supernatant, human IL-1β, IL-6, and TNF-α ELISA Duoset kits were purchased from R&D and used according to the manufacturer’s protocol.

### Statistical analysis

The results are presented as the mean ± SD where applicable. Student’s *t* tests were used to compare pairs of independent groups. For all tests, values of *p* < 0.05 were considered statistically significant.

## Results

### P.g LPS induces the activation of multiple kinases in HGFs

**Figure 1:**
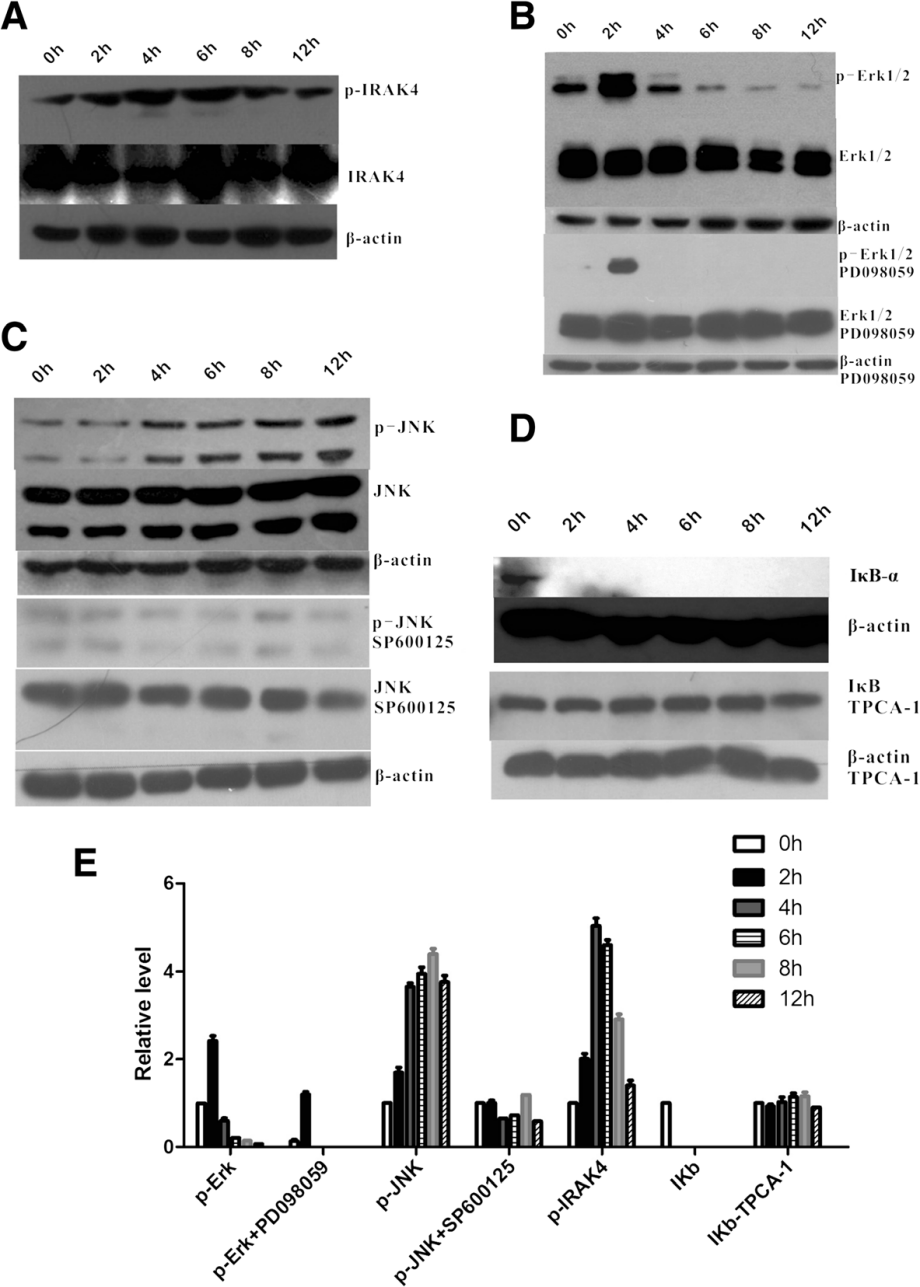
**Multiple kinases are induced in** ***P.g*** **LPS-treated HGFs.** HGFs from donors were cultured for at least 4 passages. After stimulating with *P.g* LPS (concentration 1 μg/ml) for different indicated time, HGFs were counted and harvested, and phosphorylation and expression levels of TLR4 downstream kinases such as IRAK4 **(A)**, Erk1/2 **(B)**, JNK**(C)**, and IκB-α **(D)** were analysed by monoclonal and polyclonal antibodies. The concentrations of TPCA-1 (an IKK-2 inhibitor), PD98059 (a MEK-1/2 inhibitor), and SP600125 (a JNK-1/2 inhibitor) are all 1 μM. **(E)** Quantitative assay of **(A)**-**(D)**, data are shown as mean ± SD of three independent experiments.

After *P.g* LPS treatment, the HGFs produced inflammatory cytokines including IL-1β, IL-6, and TNF-α. These cytokines secretion are all depend on TLR4 and its downstream kinases [14]. Prior to using specific inhibitors to block these kinases, we first analysed the activation states of these kinases after *P.*g LPS treatment. We used 1 μg/ml *P.*g LPS to stimulate the HGFs, harvested the cells between 0 and 12 h, and detected the expression and phosphorylation levels of the IRAK4, JNK, Erk and IκB-α kinases by western blot. IRAK4 was activated as early as 2 h after exposure to *P.*g LPS, then activation peaked at 6 h, and IRAK4 was subsequently dephosphorylated (Figure 1A). Similar to IRAK4, Erk1/2 also reached its highest activation level 2 h after *P.*g LPS stimulation and returned to the unstimulated level at 4 h (Figure 1B). There is slight activation of JNK, even without the stimulation of *P.*g LPS, same as reported in other fibroblast research [15]. This type of constitutive phosphorylation of JNK may be caused by serum in the cell culture medium or other reason unknown, but we still found obvious increase of JNK activation after *P.*g LPS stimulation. This phosphorylation of JNK reaches its peak in 6–8 h, and keeps in high level after 12 h (Figure 1C). We did not detect IκB-α expression or its phosphorylation 2 h after *P.*g LPS stimulation, which confirmed its rapid degradation and proved that NF-κB signaling is activated after that time point (Figure 1D). Taken together, these results suggest that LPS-TLR4 signal transduction activates multiple kinases in HGFs, particularly NF-κB and Erk1/2 (Figure 1E).

To further test whether selective pharmacological inhibitors were functional in HGFs, we stimulated the cells with separate inhibitors and analysed the phosphorylation levels and total amounts of their targets. As expected, based on reports of other cell lines, PD98059 (an inhibitor of Erk kinase-1/2 and MEK-1/2, which is an upstream activator of Erk-1/2) [16], SP600125 (a JNK-1/2 inhibitor) [17], and TPCA-1 (which inhibits IKK-2 and prevents the degradation of the IκB-α proteins and NF-κB signals) [18,19] worked well in HGFs (Figure 1 B-E).

### miRNA-146 level was consistent with the activation states of the NF-κB signals

**Figure 2:**
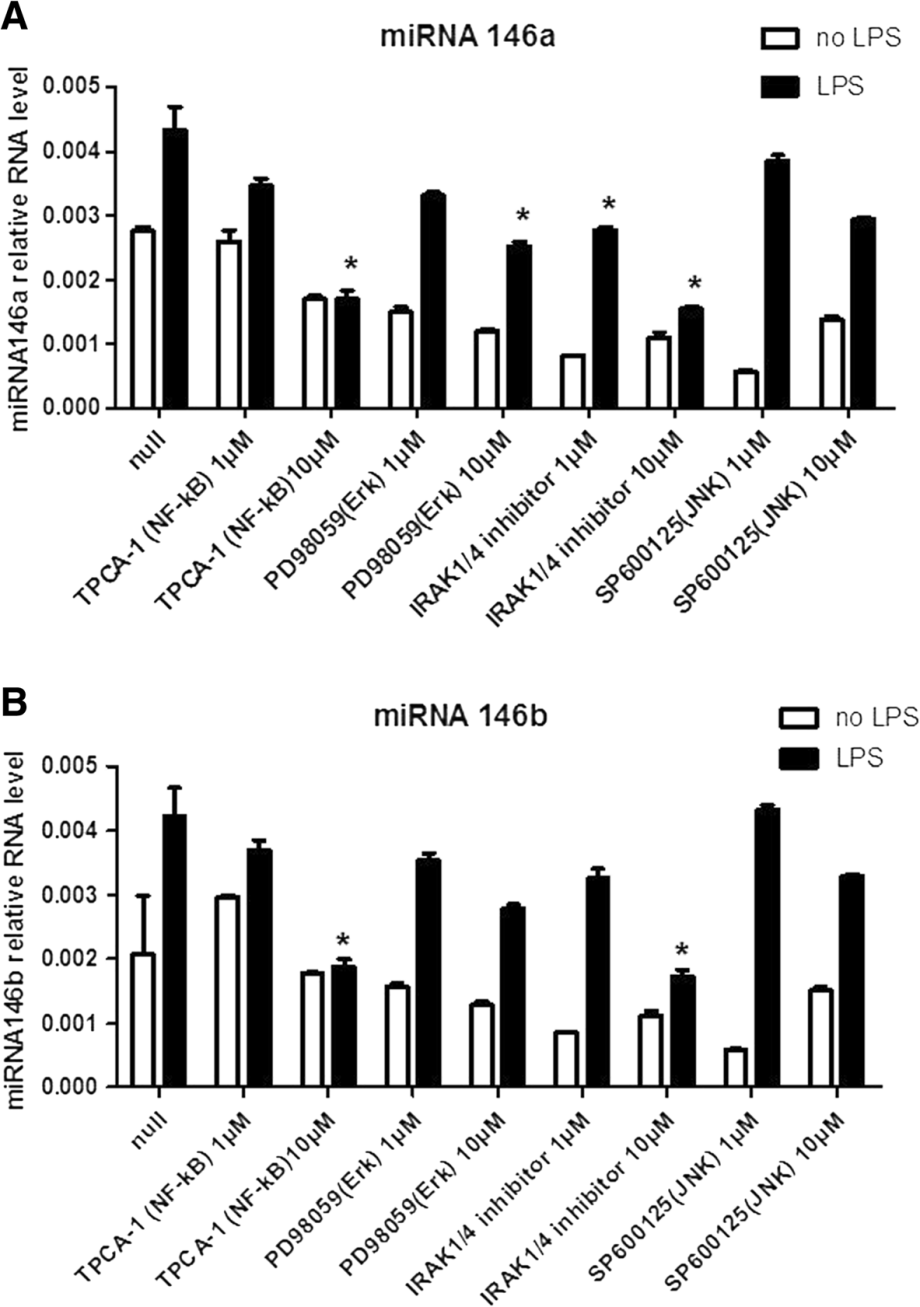
**miRNA-146 expression is down-regulated when IRAK4 and NF-κB signals are blocked.** The indicated amounts of TPCA-1 (an IKK-2 inhibitor), PD98059 (a MEK-1/2 inhibitor), IRAK1/4 inhibitor and SP600125 (a JNK-1/2 inhibitor) were added to the HGF cultures with or without *P.g* LPS (1 μg/ml) stimulation for 24 h. Next, total RNA was purified, and the miR-146a **(A)** and miR-146b **(B)** levels were analysed via real-time PCR. The results are expressed as the mean ± SD of three independent experiments. *p <0.05 versus *P.g* LPS-treated no-activation controls.

**Figure 3:**
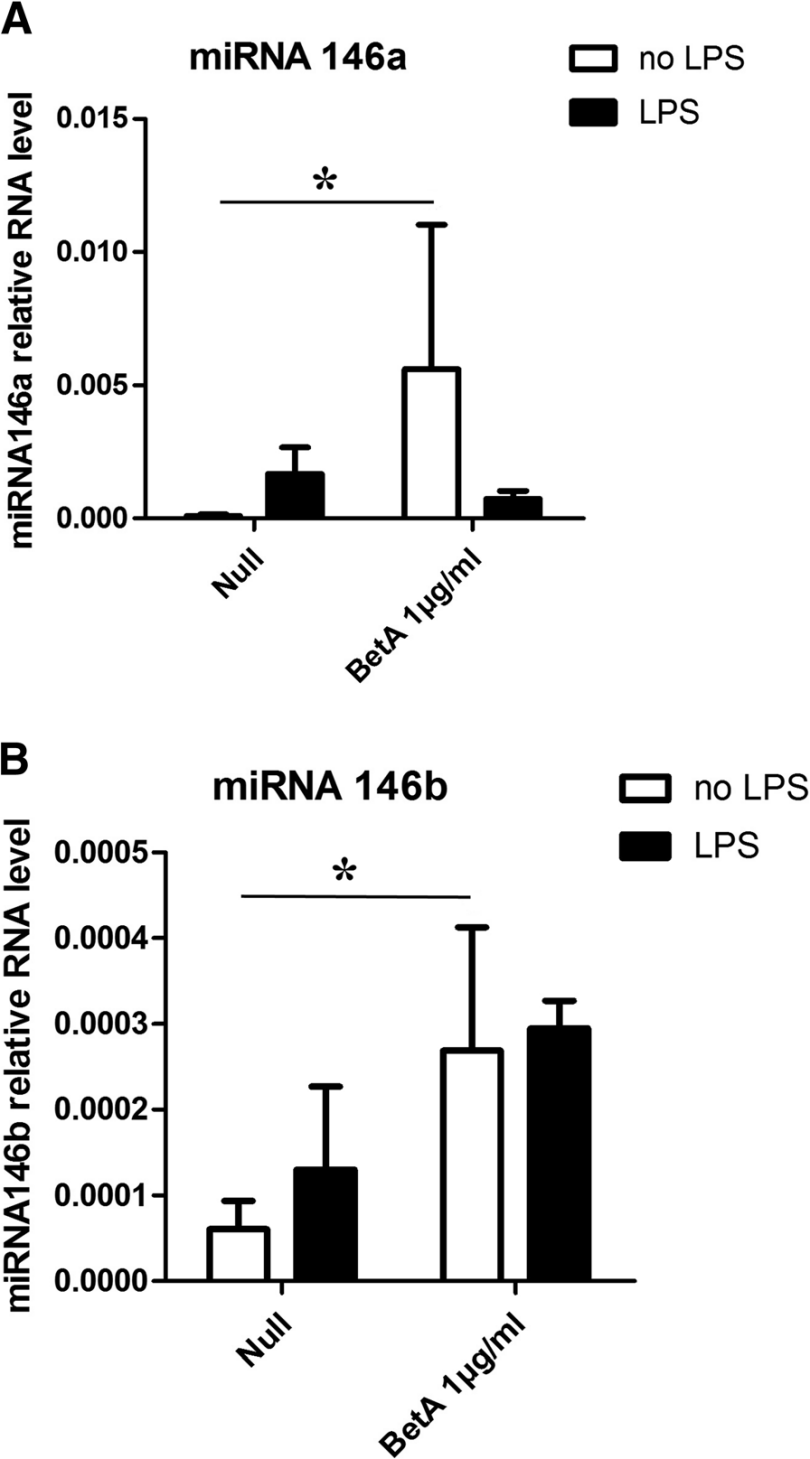
**miRNA-146 is up-regulated by the activation of NF-κB signals.** HGFs were treated with 1 μg/ml BetA (an NF-κB activator) for 24 h and then stimulated with or without *P.*g LPS (1 μg/ml) for another 24 h. Total RNA was extracted, and the miR-146a **(A)** and miR-146b **(B)** levels were analysed via real-time PCR. The results are expressed as the mean ± SD of three independent experiments. *p <0.05 versus *P.g* LPS-treated no-activation controls.

To explore whether miRNA-146 expression is regulated by these kinases during activation of LPS-TLR4, we further used selective pharmacological inhibitors, such as the IRAK1/4 inhibitor [20], TPCA-1, SP600125 and PD98059 to block the overall downstream signal pathway or specific pathway of TLR4. In our previous report [12], we found that HGFs strongly express miR-146a and miR-146b 24 h after *P.*g LPS treatment and their expression levels depend on TLR4-IRAK1/4 signals. To further analyse the precise mechanisms of miR-146 expression, we treated the HGFs with different kinase inhibitors at the indicated concentrations and analysed miRNA expression levels. Treatment with a high concentration (10 μM) of IRAK1/4 inhibitor or the NF-κB inhibitor TPCA-1 led to a reduction of miR-146 expression compare to control. However, usage of SP600125 or PD98059, blocking JNK or Erk signalling pathways respectively, only weakly influenced miR-146a (Figure 2A) and miR-146b (Figure 2B) levels. These findings indicate that miR-146 expression is mainly controlled by factors downstream of NF-κB and IRAK1/4 other than JNK or Erk signals. Moreover, we also used betulinic acid (BetA), which activates NF-κB by increasing IKK activity and IκB-α degradation [21]. After 24 h of treatment with BetA, the levels of miR-146 expression in the HGFs were greater than those of the controls regardless of the *P.*g LPS stimulation condition (Figure 3). Taken together, these results imply that miR-146 expression is under the regulation of NF-κB signals.

### *P.*g *LPS-induced inflammatory cytokine expression is affected by IRAK1/4 and NF-κB inhibitor*

**Figure 4:**
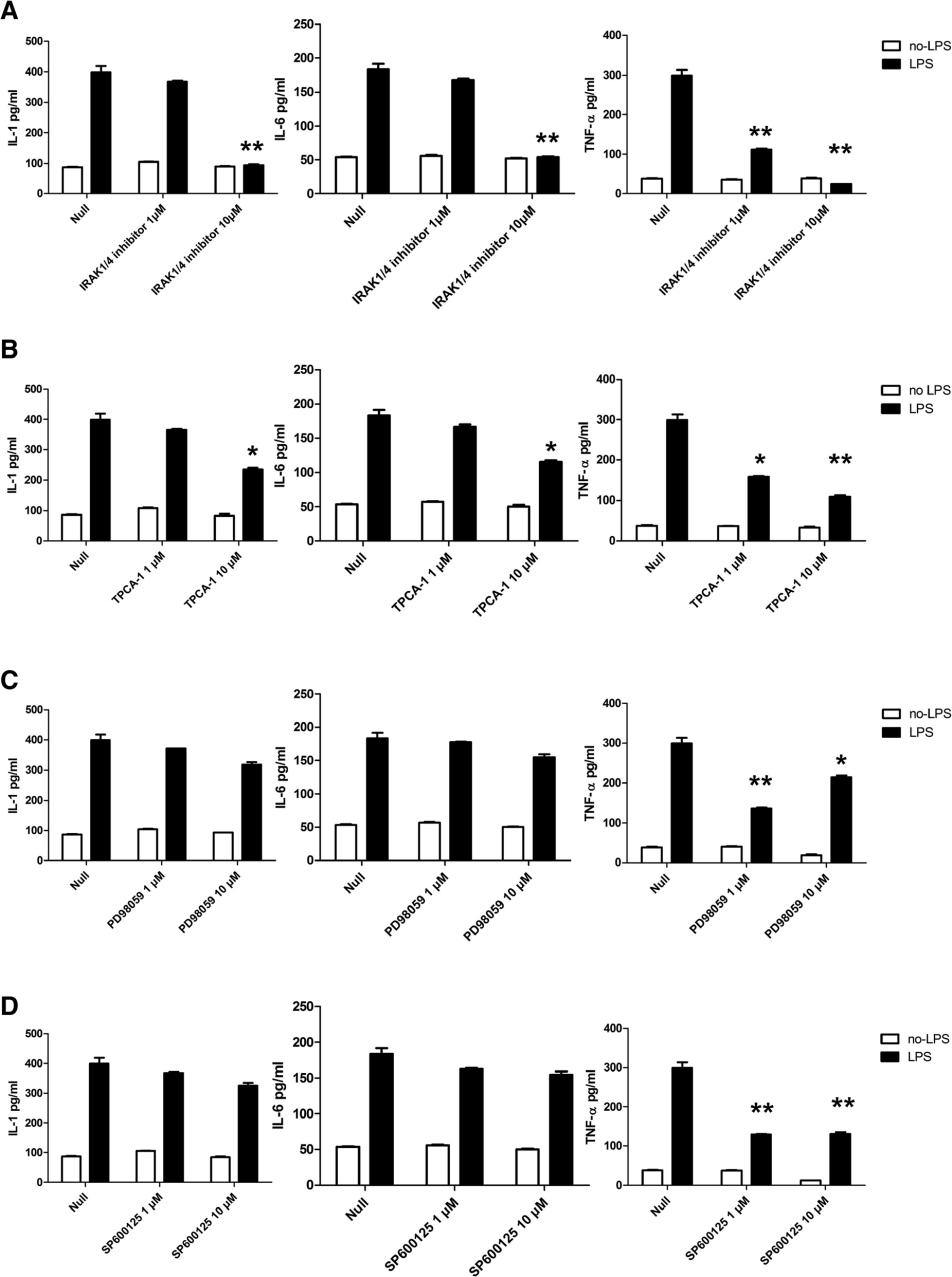
**Cytokine expression in HGFs is under the regulation of signals downstream of TLR4.** HGFs were cultured with inhibitors at the indicated concentrations with or without *P.*g LPS for 24 h. The supernatants were harvested and *P.*g LPS-induced cytokine levels were measured with ELISA to identify the contributions of IRAK4 **(A)**, NF-κB **(B)**, Erk **(C)**, and JNK **(D)** to inflammatory cytokines production. The results are expressed as the mean ± SD of three independent experiments. *p <0.05, and **p <0.01 versus *P.*g LPS-treated no-activation controls.

miR-146 level is not up-regulated under *P.*g LPS-TLR4 stimulation when using NF-κB and IRAK1/4 inhibitor (Figure 2). And key result of the *P.*g LPS-TLR4 signals in HGFs is the production of numerous inflammatory cytokines that induce inflammation. Next, we want to explore whether cytokine secretion is also affected as miR-146 by IRAK1/4 and NF-κB inhibitors. In our earlier research [12], we found that high levels of IL-1β, IL-6 and TNF-α are secreted by HGFs after *P.*g LPS treatment. Cytokine secretion is further elevated when miR-146 is inhibited, which suggests that miR-146 plays a key role in the modulation of LPS-induced cytokine production. Thus, we next used specific pharmacological inhibitors to uncover the specific mechanisms of the expression of inflammatory cytokines in *P.*g LPS-treated HGFs. The indicated concentrations of inhibitors were added to the HGF culture media with or without *P.*g LPS simulation. Twenty-four hours later, the supernatants were harvested, and the cytokines levels were analysed by ELISA. As expected, IRAK1/4 inhibited the expression of most of the cytokines at the 10 μM concentration, and TNF-α was the most sensitive and its level decreased even under the low concentration (1 μM) of IRAK1/4 inhibitor (Figure 4A). Interestingly, TPCA-1 partially mimicked the effects of IRAK1/4; it substantially reduced IL-1β and IL-6 levels at the 10 μM concentration, and inhibited TNF-α at both the low and high concentrations (Figure 4B). In contrast, PD98059 and SP600125 only weakly influenced IL-1β and IL-6 levels at either the high or low concentrations, but both inhibitors down-regulated TNF-α levels (Figure 4C, 4D). In summary, these data indicate that HGF cytokine expression after *P.*g LPS stimulation is subject to complicated control mechanisms; IL-1β and IL-6 depend primarily on NF-κB signals, and TNF-α relies on both the NF-κB and AP-1 pathways.

### Direct targeting of the promoters of miR-146 by NF-κB

**Figure 5:**
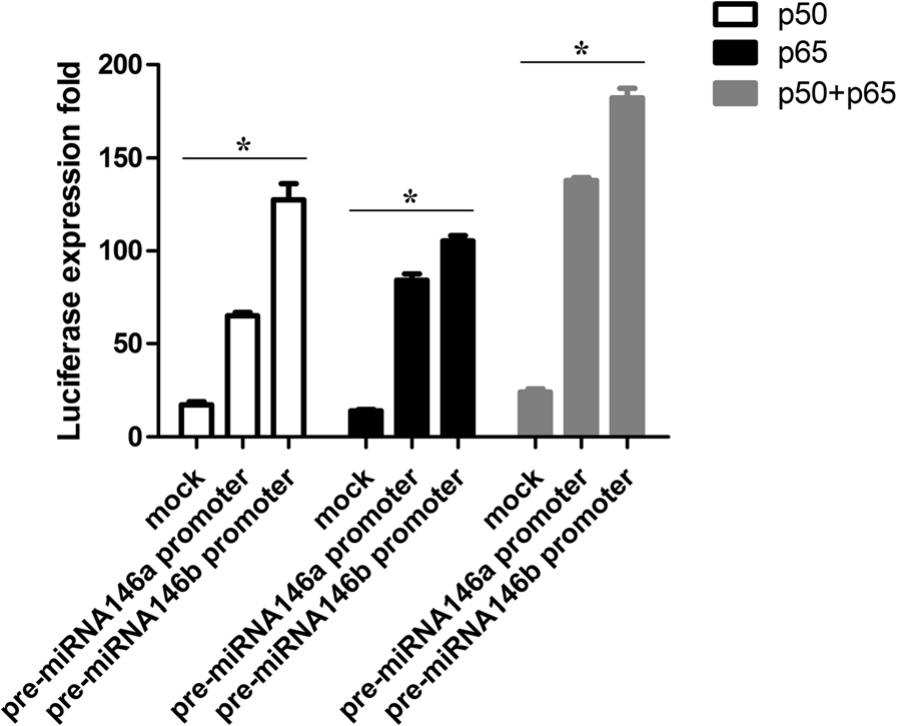
**NF-κB subunits directly bind to the miR-146 promoter.** We cloned the miR-146 2 kb promoter region into the 5’ site of the luc2 reporter gene on the pGL4 plasmid. We co-transfected 200 ng of the reporter plasmid, 20 ng pRL-TK-Renilla-luciferase and 100 ng of p50/p65 pcDNA3.0 plasmid into the HGFs. Luciferase activity was measured using the Dual-Luciferase Reporter Assay System according to the manufacturer’s instructions. The expression levels are presented as the mean ± SD. *p <0.05 versus mock control.

It has been reported that both the miR-146a and miR-146b genes have putative NF-κB binding sites in their promoter loci and that the promoter constructs within these loci can be activated in luciferase assays after TLR4 stimulation [9]; these findings provide strong evidence that NF-κB directly controls the expression of miR-146. To confirm whether same situation exists in HGFs, we constructed separate plasmids containing either miR-146a or miR-146b 5’ promoter sequence in upstream of the luciferase reporter gene. Overexpression of p50 or p65 (common members of the NF-κB family) revealed that either p50 or p65 can activate the promoter of miR-146. Furthermore, co-transfection revealed that p50 and p65 have additive effects on the 5’ promoters of miR-146a and miR-146b (Figure 5). These results prove that the promoter of miR-146 is the target of NF-κB in HGFs.

## Discussion

Toll-like receptors are broadly distributed on divergent immune cells, function as primary sensors of alien PAMPs (such as LPS), and trigger the activation of signal cascades and the production of inflammatory cytokines [22]. However, excessive activation of the TLR signalling pathway leads to immune disorders such as autoimmune or chronic inflammatory disorders [23]. Therefore, TLR signalling and its consequences must be under tight and precise negative regulation to prevent over-response during efficiently clearing the pathogens. Thus far, multiple negative responses have been found in the TLR signalling network, and these negative regulators reduce TLR signals via multiple mechanisms, such as degradation by ubiquitination, competitive binding in the form of alternative splicing [24], and, most importantly and recently discovered, the down-regulation of gene expression via miRNA targeting [25].

Some miRNAs play key roles in regulating inflammatory responses or tolerance. miRNA-21, which is triggered by LPS-induced NF-κB signals in human peripheral blood mononuclear cells, targets peripheral blood CD4^+^ cells and thus positively influences IL-10 production. Decreases in miR-21 due to transfection with miR-21 antisense oligonucleotides lead to strong increases in NF-κB signals and IL-10 production; these findings prove that miR-21 is the fundamental molecule in the NF-κB negative regulation loop [26]. However, in epithelial cell lines, miR-21 is activated by IL-6 and STAT3 and targets PTEN, which leads to increased NF-κB activity and tumour growth [27]. These results imply that the activation and function of miRNAs vary across cell conditions and that the precise mechanisms controlled by miRNAs are complicated.

miR-146a expression was found to be an NF-κB-dependent via *in vitro* reporter assays in the breast cancer cell line [28]. In macrophages, the inhibition of NF-κB by pyrrolidinecarbodithoic acid (PDTC), which is a chemically synthesised inhibitor of NF-κB, also impairs VSV-induced miR-146a expression [11]. However, there are no reports regarding whether NF-κB is responsible for the expression of miR-146b. In HGFs, Erk1/2 activation and the subsequent activation of AP-1 have been reported to occur after LPS stimulation [4,5], but the overall relationship between the miRNA negative feedback loop and the LPS signalling cascades remains to be explored.

Here, we found that TLR4 signalling initiated a rapid and continuous activation of NF-κB activation and a much shorter-termed Erk1/2 phosphorylation in HGFs. We also observed that both miR-146a and miR-146b levels were dramatically reduced by blockade of the NF-κB signal but not by inhibition of JNK or Erk1/2. As expected, cytokine production was primarily dependent on NF-κB, and thus NF-κB signalling requires further downstream negative regulations to prevent over-responding to antigens. Our results show that both miR-146a and miR-146b are downstream targets of NF-κB but not AP-1. Thus, in HGFs, NF-κB has a greater role in the production of inflammatory cytokines than AP-1 does, and miR-146 may be one of the key downstream negative regulators of NF-κB.

miR-146a and miR-146b are highly expressed in the metastatic human breast cancer cell line MDA-MB-231 and negatively regulate NF-κB activity by targeting the 3’ UTRs of IRAK1 and TRAF6. miR-146a/b overexpression in MDA-MB-231 cells leads to markedly impaired invasion and migration capacity compared to controls, and this finding proves that miR-146 suppresses NF-κB activity via a reduction of metastatic potential [28]. Furthermore, miR-146 levels are significantly up-regulated by breast cancer metastasis-suppressor 1 (BRMS1), a gene that affects multiple steps in the metastatic cascade, which leads to a suppression of metastasis of ~90% in breast carcinomas [29]. In HGFs, we found that TNF-α is more sensitive to pharmacological inhibitors. These results imply that miR-146 has a fundamental function in controlling cancer-related signals and in inhibiting inflammatory cytokines.

Although only BetA or *P.g* LPS stimulation can up-regulate miR-146a and miR146b in HGFs, our data showed different express patterns of miR-146a and miR-146b under both BetA and *P.g* LPS stimulation. miR-146a and miR146b share similar sequence and function, the genomic loci of miR-146a and miR146b is on human chromosomes 5 and 10 respectively, far away from each other, which implies their transcription may under different regulation. This gave us hint that there may be other miR-146 regulation mechanisms under LPS stimulation, other than NF-κB, which we will study in our future work.

miRNAs are regulated by precise mechanisms and are thought to target multiple mRNAs to regulate gene expression. As the number of miRNAs is much smaller than the number of coding genes, single miRNAs may regulate different genes in different conditions. Our research showed that miR-146 is highly regulated in *P.*g LPS-treated HGFs and negatively controls TLR4 signalling by targeting IRAK1 but not TRAF6 [12]. We also found that NF-κB but not AP-1 is responsible for the production of both miR-146a and miR-146b. Moreover, miR-146 inhibits NF-κB signals and limits immune responses to appropriate levels. It is probable that other activators in the NF-κB signalling pathway are targets of miR-146. This presumption will lead us to work of seeking to reveal the entire set of functions of miR-146 in the periodontal innate immune response.
